# Silver quantum dots in plasmonic photocatalysis: linking quantum confinement to structure–function relationships

**DOI:** 10.1039/d6ra05311d

**Published:** 2026-07-15

**Authors:** Asokan Vasudevan, Suleiman Ibrahim Mohammad, Zukhra Yakhshieva, Ibrokhim Sapaev, Man Mohan Shukla, Monika Verma, Pankaj Tripathi, Tawfeeq Alghazali, Taraneh Hieunaz Chavoushi

**Affiliations:** a Faculty of Business and Communications, INTI International University 71800 Negeri Sembilan Malaysia; b Department of Business Administration, Business School, Al Al-Bayt University Mafraq 25113 Jordan; c Research Follower, INTI International University 71800 Negeri Sembilan Malaysia; d Chemistry Department, Jizzakh State Pedagogical University Jizzakh City Uzbekistan; e Head of the Department, Physics and Chemistry, Tashkent Institute of Irrigation and Agricultural Mechanization Engineers, National Research University Tashkent Uzbekistan; f Scientific Researcher of the University of Tashkent for Applied Science Uzbekistan; g School of Engineering, Central Asian University Tashkent 111221 Uzbekistan; h Western Caspian University, Scientific Researcher Baku Azerbaijan; i Department of Computer Science and Engineering, Pranveer Singh Institute of Technology Kanpur UP India; j Department of Chemistry, University Institute of Sciences, Chandigarh University Mohali Punjab India; k Department of Physics, Sharda University Knowledge Park III Greater Noida India; l English Department, The Islamic University in Najaf Iraq; m Young Researchers and Elite Club, Tehran Branch, Islamic Azad University Tehran Iran thanhhieunchauu@gmail.com

## Abstract

Silver quantum dots (AgQDs) have emerged as a distinctive class of plasmonic nanomaterials that bridge the gap between conventional metallic nanoparticles and quantum-confined systems. Their unique electronic structure, surface atomic organization, and size-dependent optical properties have attracted increasing attention for photocatalytic applications. This review critically examines the fundamental structure–function relationships governing AgQD-enabled plasmonic photocatalysis, with particular emphasis on the transition from metallic behavior to quantum confinement and its implications for photocatalytic performance. Recent advances in understanding size-dependent electronic states, surface heterogeneity, plasmonic responses at the quantum limit, charge-carrier dynamics, and interfacial energy-transfer pathways are systematically discussed. The review further analyzes how these properties influence key photocatalytic processes, including hydrogen evolution, CO_2_ reduction, environmental remediation, and photoelectrochemical conversion. Rather than considering AgQDs as simple plasmonic additives, emerging evidence highlights their multifunctional roles in light harvesting, charge management, interfacial modulation, and catalytic activation. By integrating mechanistic insights with application-oriented developments, this review identifies the key design principles linking quantum confinement to photocatalytic function. Finally, unresolved challenges, including mechanistic ambiguity, atomic-level control, interface engineering, and scalability, are discussed to outline future directions for the rational design of next-generation AgQD photocatalysts.

## Abstract 2

Silver quantum dots bridge plasmonic and quantum-confined regimes, enabling tunable charge separation and interfacial electron transfer. This review highlights structure–function relationships driving photocatalysis and future design strategies.

## Graphical abstract

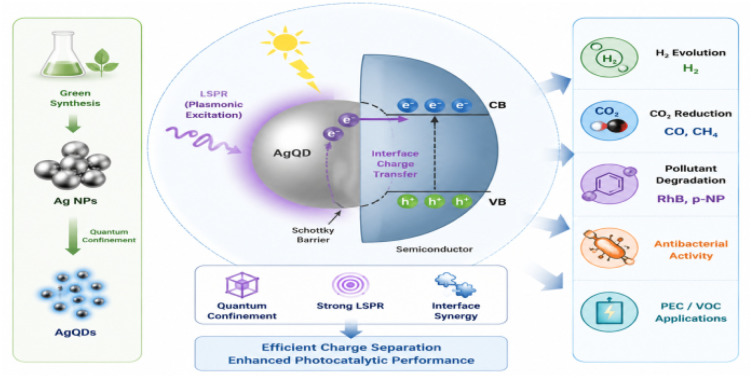

## 1. Introduction

Photocatalysis has emerged as one of the most promising strategies for addressing global challenges associated with sustainable energy production and environmental remediation. By utilizing solar energy to drive chemical transformations, photocatalytic systems offer attractive pathways for hydrogen generation, carbon dioxide conversion, pollutant degradation, and water purification.^1–4^ Despite significant progress in semiconductor-based photocatalysts, practical implementation remains limited by inefficient visible-light utilization, rapid charge-carrier recombination, and insufficient control over interfacial charge-transfer processes. These limitations have stimulated increasing interest in advanced nanostructured materials capable of improving light–matter interaction and catalytic efficiency.^5–8^

Among various candidates, plasmonic noble-metal nanostructures—particularly silver-based systems—have attracted considerable attention due to their strong electromagnetic field enhancement, high conductivity, and tunable optical properties.^9–15^ In recent years, a new class of ultrasmall silver-based materials, referred to as silver quantum dots (AgQDs), has emerged at the intersection of plasmonics and quantum-confined systems. These materials introduce unique physicochemical characteristics that extend beyond conventional plasmonic nanoparticles, enabling new opportunities for manipulating light absorption, charge dynamics, and surface reactivity.

Importantly, AgQDs differ fundamentally from larger silver nanoparticles in that their properties are not solely governed by classical plasmonic theory but are also influenced by size-dependent electronic discretization, surface atomic structure, and ligand-mediated effects. This hybrid nature positions AgQDs as multifunctional platforms capable of simultaneously participating in light harvesting, charge modulation, and catalytic activation processes. As a result, they have been increasingly explored in hydrogen evolution, CO_2_ reduction, environmental remediation, photoelectrochemical systems, and antimicrobial applications.^16–19^

Despite this progress, the field remains highly fragmented. Most studies report enhanced catalytic performance without establishing unified mechanistic relationships between size-dependent electronic structure, plasmonic behavior, and interfacial charge-transfer processes.^20–23^ In particular, the lack of clear distinction between quantum confinement effects and general nanoscale phenomena has led to inconsistent mechanistic interpretations across different studies.

Accordingly, this review aims to provide a comprehensive and mechanistically consistent framework for AgQD-based photocatalysis. By integrating recent advances across hydrogen evolution, CO_2_ conversion, environmental remediation, and antibacterial applications, we systematically analyze structure–function relationships governing AgQD behavior. Special emphasis is placed on the interplay between quantum confinement, plasmonic functionality, and interfacial charge dynamics, with the goal of establishing unified design principles for next-generation silver-based photocatalysts.

## 2. AgQDs beyond nanoscale metals: structure–property relationships governing plasmonic functionality

### 2.1. Size-dependent electronic structure: the transition from metallic nanoparticles to quantum-confined silver domains

The reduction of silver nanostructures from bulk-like nanoparticles to ultrasmall domains leads to a continuous evolution of electronic structure that cannot be fully described by classical free-electron models. In larger silver nanoparticles, electronic states are quasi-continuous and optical behavior is dominated by collective electron oscillations. However, as the particle size approaches the sub-nanometer to few-nanometer regime, electronic confinement effects become increasingly significant, leading to progressive modification of energy-level distribution and charge localization behavior.^24,25^

In this regime, AgQDs do not represent a purely metallic or purely molecular system but instead occupy an intermediate electronic state in which delocalized and discretized electronic features coexist. This hybridization results in size-sensitive modulation of carrier dynamics, excitation pathways, and electronic density distribution. Importantly, this evolution is not governed solely by geometric size but is strongly influenced by atomic arrangement, surface coordination, and ligand-induced perturbations, which collectively determine the effective electronic landscape.^26–28^

From a design perspective, controlling the degree of electronic confinement provides a route for tuning the intrinsic electronic properties of AgQDs prior to catalytic application. However, significant variability in reported size-dependent behavior highlights the lack of a universal threshold for metallic-to-confined transition, emphasizing the need for a structure–sensitive interpretation of electronic properties.^29,30^ This electronic framework forms the basis for understanding the optical response discussed in the following subsection.

**Fig. 1:**
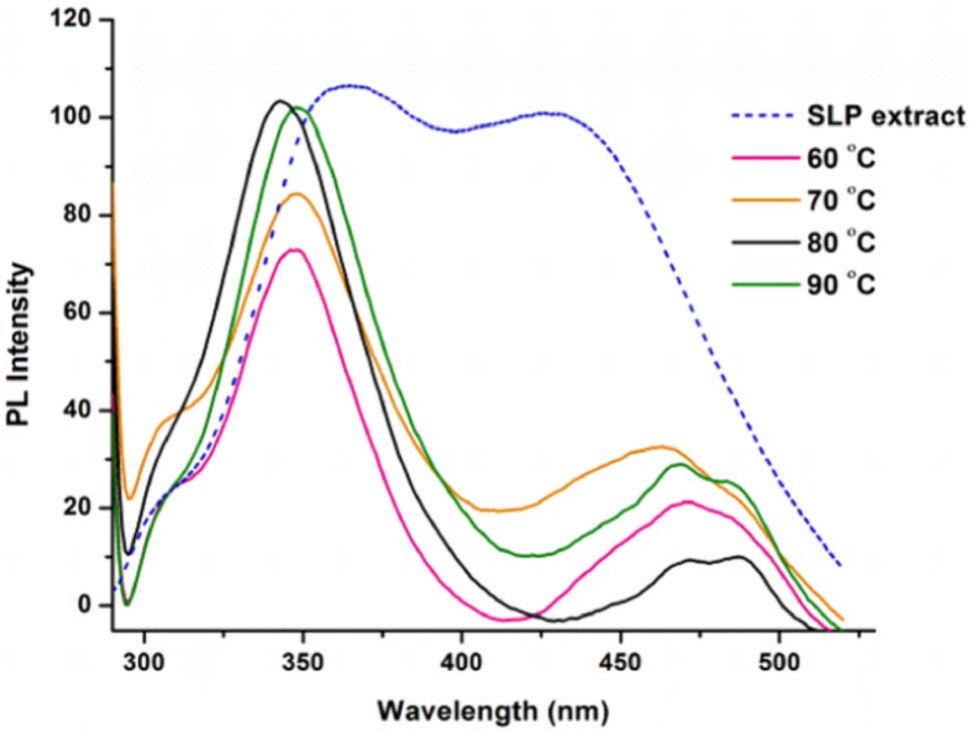
Photoluminescence spectra of AgQDs synthesized at different reaction temperatures, illustrating size-dependent electronic transitions and quantum-confinement effects. Reproduced with permission from ref. 27. © 2021 Elsevier B.V.

The evolution of the electronic structure in AgQDs can be inferred from their photoluminescence behavior, which reflects the nature of electronic transitions within quantum-confined silver domains. As shown in Fig. 1, the PL spectra exhibit emission bands in the 460–480 nm region that originate from radiative recombination between excited sp-band electrons and d-band holes. Variations in emission intensity with reaction temperature indicate changes in the electronic states of the AgQDs, suggesting that synthesis-dependent structural modifications influence carrier relaxation pathways and excited-state dynamics. Notably, the gradual blue shift of the emission peaks with increasing reaction temperature is consistent with a reduction in particle size and the strengthening of quantum confinement effects.

Such behavior reflects the progressive discretization of electronic energy levels that accompanies the transition from larger metallic nanostructures toward ultrasmall quantum-confined silver domains. Furthermore, the quenching and spectral shift observed relative to the precursor extract indicate electronic coupling between surface-bound biomolecules and the AgQD core, highlighting the sensitivity of confined electronic states to the local chemical environment. These observations collectively support the concept that the electronic architecture of AgQDs is strongly governed by size-dependent quantum effects and surface-mediated perturbations.

### 2.2. Surface atomic organization and electronic heterogeneity in AgQDs

As AgQDs approach atomic precision, surface atoms become the dominant structural component rather than a peripheral feature. In contrast to larger metallic nanoparticles, where bulk atoms dictate most electronic characteristics, a substantial fraction of atoms in AgQDs resides at surfaces, edges, and low-coordination sites. Consequently, electronic properties become highly sensitive to local atomic arrangements, generating pronounced electronic heterogeneity across the nanostructure. This shift fundamentally alters how structure–property relationships should be interpreted, since average compositional descriptors often fail to capture the complexity of localized electronic environments.

Recent advances indicate that surface atomic organization influences electronic behavior through multiple interconnected mechanisms.^31–33^ Variations in coordination number modify local electron density, while surface reconstruction may generate energetically distinct active domains within the same QD. Furthermore, atomic vacancies, lattice distortions, and undercoordinated silver atoms can introduce localized electronic states that significantly perturb charge distribution. These structural features are particularly important because their influence extends beyond geometric considerations and directly affects the energetic landscape of electronic excitations. As a result, two QDs with comparable size distributions may exhibit markedly different electronic characteristics due to differences in atomic ordering.

A critical challenge in the field arises from the limited ability of conventional characterization techniques to resolve dynamic surface structures under realistic operating conditions. Most structural models rely on *ex situ* observations, whereas surface atoms frequently undergo reconstruction in response to environmental interactions. This discrepancy contributes to persistent uncertainties regarding the true origins of many reported electronic phenomena. Moreover, the role of ligand–surface interactions remains insufficiently understood, despite growing evidence that surface chemistry can alter electronic states as strongly as intrinsic structural defects.^34–36^ These observations collectively suggest that surface atomic architecture should be treated as an independent design variable rather than a secondary consequence of synthesis. Establishing direct links between atomic organization and electronic heterogeneity is therefore essential before examining how these electronic characteristics manifest in the optical response of AgQDs.

### 2.3. Optical response of AgQDs: plasmonic behavior at the quantum limit

The optical response of AgQDs in the quantum regime deviates significantly from classical localized surface plasmon resonance (LSPR) theory. In ultrasmall silver domains, electron scattering effects, finite-state occupation, and partial energy-level discretization collectively modify the nature of optical excitations. As a result, optical behavior arises from the interplay between collective plasmonic oscillations and quantum-confined electronic transitions rather than from a purely classical plasmonic mechanism alone.^37–39^

Unlike conventional nanoparticles, plasmonic features in AgQDs do not vanish abruptly with size reduction. Instead, they evolve gradually, often manifesting as broadened resonance peaks, increased damping, and enhanced sensitivity to local structural and chemical perturbations.^40,41^ This behavior indicates that plasmonic response in AgQDs should be interpreted as a structure-dependent phenomenon influenced by atomic-scale configuration rather than strictly by particle size.

At the same time, experimental interpretation of optical spectra in this regime remains challenging. Similar spectral features may originate from different underlying mechanisms, including plasmon-like resonances, quantum-confined transitions, or hybrid excitation modes. This mechanistic ambiguity highlights the limitations of classical–quantum separation and emphasizes the need for unified theoretical models capable of bridging electrodynamic and quantum descriptions.^42–44^ From a functional perspective, the optical response directly governs light harvesting, energy localization, and charge excitation processes in AgQDs. Therefore, understanding this hybrid optical regime is essential for rational design of plasmonic photocatalysts, which will be further connected to structure–property design principles in the next subsection.

**Fig. 2:**
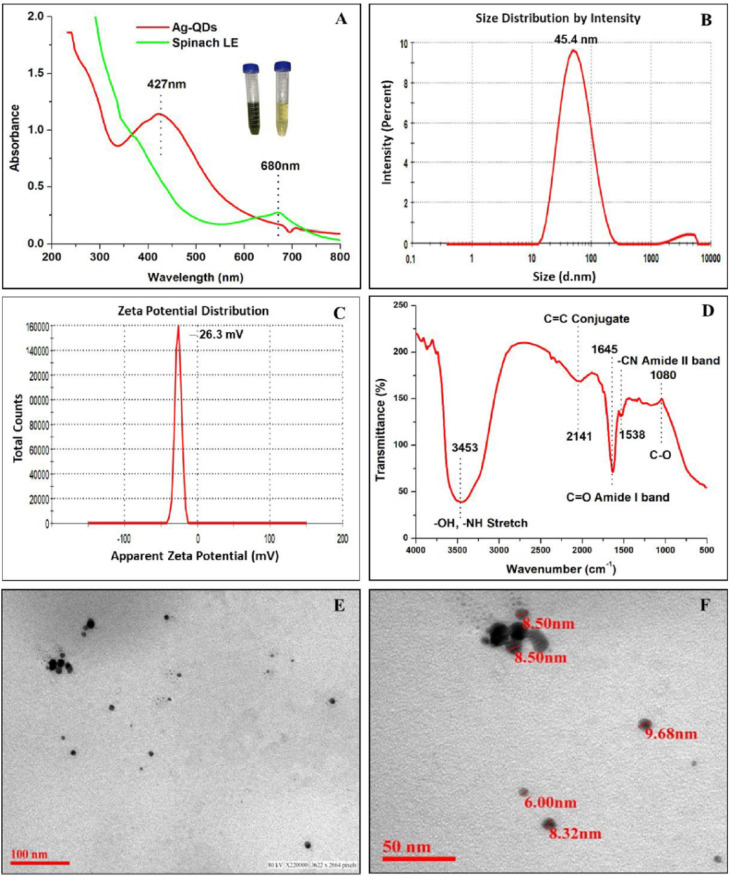
Physicochemical characterization of AgQDs showing (A) UV-vis absorption spectrum, (B) hydrodynamic size distribution, (C) zeta potential, (D) FTIR spectrum, and (E and F) TEM images illustrating particle morphology and size. Reproduced with permission from ref. 43. © 2025 Elsevier B.V.

The optical characteristics of AgQDs are strongly influenced by the interplay between particle size, surface chemistry, and electronic confinement effects. As shown in Fig. 2A, the UV-vis absorption spectrum exhibits a distinct absorption band centered at 427 nm, which is characteristic of the surface plasmon resonance (SPR) of silver nanostructures and reflects the ability of AgQDs to support collective electron oscillations despite their ultrasmall dimensions. The hydrodynamic size distribution obtained from DLS analysis (Fig. 2B) indicates a relatively narrow particle population, while TEM observations (Fig. 2E and F) reveal spherical and monodispersed AgQDs with an average core diameter of approximately 9.2 nm. Such dimensions place the particles within a regime where plasmonic behavior is increasingly affected by quantum-size effects and enhanced surface contributions. Furthermore, the negative zeta potential (Fig. 2C) confirms colloidal stability, which is essential for maintaining consistent optical properties, whereas FTIR analysis (Fig. 2D) demonstrates the presence of surface-bound functional groups originating from the plant extract. These surface ligands can modulate local electronic environments and influence plasmon damping processes, highlighting the close relationship between surface chemistry and optical response in quantum-confined silver domains.

### 2.4. Structure–property design principles for maximizing plasmonic functionality in AgQDs

The preceding discussions reveal that plasmonic functionality in AgQDs emerges from the coupled influence of quantum confinement, surface atomic architecture, and size-dependent optical evolution rather than from any single structural parameter. Consequently, the design of high-performance AgQDs requires moving beyond conventional particle-size optimization toward a multidimensional structure–property framework capable of simultaneously controlling electronic states, surface energetics, and optical excitations. Such a perspective is particularly important because structural parameters that enhance one functionality may compromise another, creating inherent design trade-offs that remain insufficiently addressed in current research.^45–47^

A recurring trend across the literature is that maximizing plasmonic performance does not necessarily coincide with achieving the smallest possible particle dimensions. Excessive size reduction may strengthen quantum confinement but can also suppress collective electron behavior through increased electronic localization and damping effects. Therefore, an optimal design window likely exists where sufficient electronic delocalization is preserved while quantum-induced modulation of optical properties remains accessible. Identifying this window represents a critical challenge for future materials engineering.^48,49^

Table 1 summarizes the key structure–property relationships governing plasmonic functionality in AgQDs. The analysis highlights that photocatalytically relevant plasmonic behavior does not arise from particle size alone but emerges from the coupled effects of quantum confinement, surface atomic organization, electronic heterogeneity, and optical evolution. These descriptors collectively determine charge-carrier dynamics, light-harvesting efficiency, and energy-transfer pathways. The framework further emphasizes that rational AgQD design requires simultaneous optimization of structural, electronic, and optical parameters to maximize functional performance in plasmonic photocatalytic systems.

**Table 1:** Three-dimensional structural–electronic–interfacial framework for AgQDs

Design dimension	Structural descriptor	Physical meaning	Influence on optical response	Impact on charge dynamics	Design implication
Electronic	QD size reduction	Quantum confinement strength	Spectral blue-shift and plasmon damping	Modified carrier generation efficiency	Tune size for optimal confinement–plasmon balance
Electronic	Energy-level discreteness	Quantization of electronic states	Emergence of hybrid optical transitions	Enhanced hot-electron formation	Control electronic quantization regime
Electronic	Density of states (DOS) reduction	Transition from metallic to molecular-like behavior	Broadened/shifted absorption features	Altered relaxation pathways	Engineer electronic density for catalytic activation
Structural	Surface coordination environment	Degree of atomic undercoordination	Modified light–matter interaction strength	Increased carrier localization	Tailor surface structure for active sites
Structural	Defect density (vacancies & disorders)	Localized electronic trap states	Resonance broadening and damping effects	Enhanced charge trapping/recombination control	Defect engineering for tuned activity
Structural	Surface reconstruction dynamics	Environment-induced atomic rearrangement	Variable optical signatures	Time-dependent carrier transport	*Operando* structural control strategies
Interfacial	Ligand–surface interaction	Electronic coupling with organic ligands	Plasmon shift and damping modulation	Interfacial charge regulation	Rational ligand design for charge tuning
Interfacial	Dielectric environment (*ε*_env_)	External screening effect	Shift in plasmon resonance energy	Modified charge separation efficiency	Control surrounding medium for tuning response
Interfacial	Support interaction	Electronic coupling with substrate	Enhanced or quenched plasmonic modes	Directional charge transfer pathways	Interface engineering for efficient extraction

Equally important is the control of atomic-scale surface organization. Surface defects, low-coordination sites, and structural heterogeneities can either enhance or destabilize plasmonic responses depending on their spatial distribution and electronic influence. This observation suggests that defect engineering should evolve from maximizing defect density toward tailoring defect functionality. In this context, atomic precision becomes more valuable than structural complexity. Rationally designed surface architectures capable of maintaining electronic coherence while introducing beneficial local perturbations may provide superior performance compared with highly defective but poorly controlled systems.

Another emerging principle involves treating optical behavior as an outcome of integrated structural design rather than an isolated property. Since electronic structure, surface organization, and optical response are intrinsically interconnected, predictive optimization requires simultaneous consideration of all three descriptors.^50–52^ The field is therefore moving toward data-driven and atomistically informed design strategies that emphasize mechanistic understanding over empirical optimization. Establishing such integrated design principles is essential for transforming AgQDs from scientifically intriguing nanostructures into functionally tunable plasmonic platforms, thereby creating the conceptual foundation for evaluating their roles in photocatalytic systems discussed in the following section.

### 2.5. Size-ligand-environment regime map for metallic, plasmonic, and quantum-confined AgQDs

To address the ambiguity in distinguishing metallic, plasmonic, and quantum-confined behavior in AgQDs, a unified conceptual regime map is introduced based on the coupled roles of particle size, electron scattering, and ligand-induced dielectric screening. In silver nanostructures, the optical response is primarily governed by the competition between electron delocalization and boundary-induced scattering. In relatively large nanoparticles, where the particle dimension (d) is significantly greater than the electron mean free path (*l*_e_ ≈ 40–50 nm in bulk Ag), collective electron oscillations dominate, leading to classical LSPR. As the particle size decreases toward a few nanometers, surface scattering becomes increasingly important, resulting in enhanced damping (*γ*) and progressive broadening of plasmonic features rather than an abrupt transition.

When the particle size approaches the electronic coherence length scale, typically in the sub-10 nm regime depending on synthesis conditions and surface chemistry, deviations from classical plasmonic behavior become more pronounced. In this transitional regime, plasmon-like oscillations coexist with emerging discrete electronic states, giving rise to hybrid optical characteristics. However, the onset of quantum confinement cannot be defined solely by a fixed size threshold, as it is strongly influenced by ligand coordination, surface atomic structure, and dielectric environment (*ε*_env_).^48–51^ Strongly binding ligands may increase electronic localization and enhance confinement-like features, whereas weakly interacting ligands tend to preserve delocalized electronic behavior and sustain plasmonic coherence even at reduced sizes.

Therefore, the optical and electronic response of AgQDs should be interpreted within a multidimensional parameter space defined by particle size (*d*), scattering rate (*γ*), electronic coherence length (*l*_e_), and environmental screening. This framework provides a physically consistent basis for rationalizing the variability reported in experimental studies, where similar-sized AgQDs may exhibit different optical signatures due to differences in surface chemistry and synthesis route. Importantly, this regime map should be considered a conceptual guideline rather than a strict boundary model, offering a unified perspective for interpreting plasmon–quantum interplay and guiding the rational design of AgQDs for photocatalytic applications.

## 3. QDs in photocatalysis: fundamental principles and functional roles

### 3.1. Light–matter interactions in QD photocatalysts: beyond conventional semiconductor excitation

The emergence of QDs as photocatalytic building blocks has fundamentally challenged traditional descriptions of photoexcitation processes in semiconductor systems. Conventional photocatalysis is typically interpreted through bulk band theory, where photon absorption generates electron–hole pairs that subsequently participate in surface reactions. However, this framework becomes increasingly inadequate as materials approach quantum-confined dimensions. In QDs, discretized electronic states, enhanced wavefunction localization, and strong surface contributions create excitation pathways that differ substantially from those operating in bulk semiconductors.

A growing body of evidence suggests that photon utilization in QD-based systems is governed by a hierarchy of competing processes extending beyond simple charge generation.^53–55^ Optical excitation may induce direct electronic transitions between quantized states, activate defect-mediated pathways, or trigger collective electronic responses depending on the structural characteristics of the QDs. Consequently, light absorption efficiency alone is often a poor predictor of photocatalytic functionality. Materials displaying comparable optical absorption may exhibit dramatically different catalytic behavior due to variations in excited-state dynamics.

An important trend in recent research is the shift from static descriptions of electronic structures toward dynamic interpretations of photoexcited states. Rather than viewing excited carriers as isolated entities, contemporary models increasingly emphasize the coupling between electronic transitions, lattice dynamics, and interfacial interactions. Such coupling influences carrier relaxation pathways, excitation lifetimes, and energy dissipation mechanisms. Yet, many mechanistic interpretations continue to rely on oversimplified band diagrams that fail to capture the complexity of quantum-confined systems.^56–58^ These limitations highlight a critical knowledge gap in the field. The challenge is no longer identifying whether QDs absorb light efficiently, but understanding how absorbed photon energy is redistributed among competing electronic processes. Establishing this mechanistic foundation is essential for evaluating the fate of photogenerated charge carriers, which constitutes the next critical step in QD-mediated photocatalysis.

### 3.2. Charge carrier dynamics in quantum-confined systems: generation, separation, and utilization

The photocatalytic performance of QDs is ultimately determined not by photon absorption itself but by the subsequent evolution of photogenerated charge carriers. In quantum-confined systems, carrier dynamics differ fundamentally from those observed in conventional semiconductors because spatial confinement modifies both energetic distributions and carrier interactions. As a result, charge generation, separation, migration, and recombination become strongly interconnected phenomena that cannot be analyzed independently. One of the most significant consequences of quantum confinement is the alteration of carrier relaxation pathways. Reduced dimensionality changes the density of available electronic states, thereby influencing how rapidly excited carriers lose energy. While prolonged carrier lifetimes are frequently considered beneficial for photocatalysis, this assumption is not universally valid.^59–61^ Extended lifetimes do not necessarily translate into improved catalytic performance if charge extraction pathways remain inefficient. This distinction underscores the importance of evaluating carrier utilization rather than relying exclusively on lifetime measurements.

**Fig. 3:**
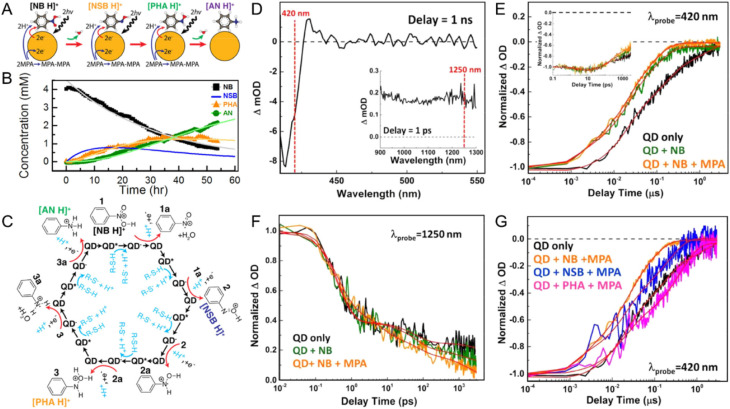
(A–C) Photocatalytic reduction pathway and catalytic cycle of nitrobenzene over CdS QDs, and (D–G) transient absorption spectra and carrier dynamics revealing charge separation and electron-transfer processes in quantum-confined photocatalysts. Reproduced with permission from ref. 60. © American Chemical Society.

The photocatalytic performance of quantum-confined systems is strongly governed by the generation, separation, and utilization of photogenerated charge carriers. As illustrated in Fig. 3A–C, CdS QDs mediate the multistep photoreduction of nitrobenzene through successive proton-coupled electron transfer processes, demonstrating how individual photogenerated electrons can be efficiently utilized in sequential catalytic transformations. The concentration profiles shown in Fig. 3B and the proposed catalytic cycle in Fig. 3C further highlight the controlled evolution of reaction intermediates, reflecting the effective management of charge-transfer events during photocatalysis. More direct insight into carrier dynamics is provided by the transient absorption measurements presented in Fig. 3D–G. The transient absorption spectra (Fig. 3D) reveal the formation of photoexcited states following light absorption, whereas the kinetic traces monitored at different probe wavelengths (Fig. 3E–G) track the temporal evolution of electrons and holes during photocatalysis. These measurements indicate rapid hole extraction by the sacrificial donor followed by electron transfer from the QDs to adsorbed reactant molecules, thereby suppressing charge recombination and extending carrier utilization. The observed dependence of electron-transfer kinetics on the thermodynamic driving force further demonstrates that photocatalytic efficiency is determined not only by carrier generation but also by the effectiveness of charge separation and interfacial carrier consumption. Collectively, these results illustrate how quantum-confined charge-carrier dynamics govern the conversion of absorbed photon energy into productive photocatalytic reactions.

Another emerging trend involves the recognition that recombination should not be viewed solely as an undesirable loss mechanism. Recent studies indicate that different recombination channels possess distinct energetic consequences, some of which may indirectly influence catalytic selectivity or reaction kinetics. Consequently, simplistic interpretations equating lower recombination rates with superior photocatalytic performance increasingly appear insufficient. Despite substantial advances in ultrafast spectroscopy and time-resolved characterization, major uncertainties remain regarding the correlation between measured carrier dynamics and actual catalytic events. Many experimental observations are obtained under idealized conditions that differ considerably from realistic reaction environments.^62–64^ Bridging this gap requires a more integrated understanding of how charge carriers evolve across multiple temporal and spatial scales. Therefore, future progress depends on moving beyond isolated carrier metrics toward comprehensive frameworks that connect charge dynamics with interfacial processes. Such interfacial phenomena represent the critical junction between photophysics and catalysis and are discussed in the following section.

### 3.3. Interfacial energy transfer pathways in QD photocatalytic systems

The interface represents the decisive region where photophysical events are converted into catalytic functionality. Regardless of how efficiently charge carriers are generated within QDs, photocatalytic activity cannot emerge unless energy or charge is effectively transferred across material interfaces. Consequently, understanding interfacial transfer mechanisms has become a central objective in modern photocatalysis research. Traditionally, photocatalytic systems were interpreted primarily through charge-transfer models involving electron or hole migration between different components. While such descriptions remain important, they no longer capture the full complexity of QD-based architectures. Recent investigations increasingly reveal the coexistence of multiple transfer pathways, including direct charge transfer, energy transfer, excitonic coupling, and plasmon-mediated interactions.^65–67^ These mechanisms often operate simultaneously and may either cooperate or compete depending on interfacial structure and energetic alignment.

**Fig. 4:**
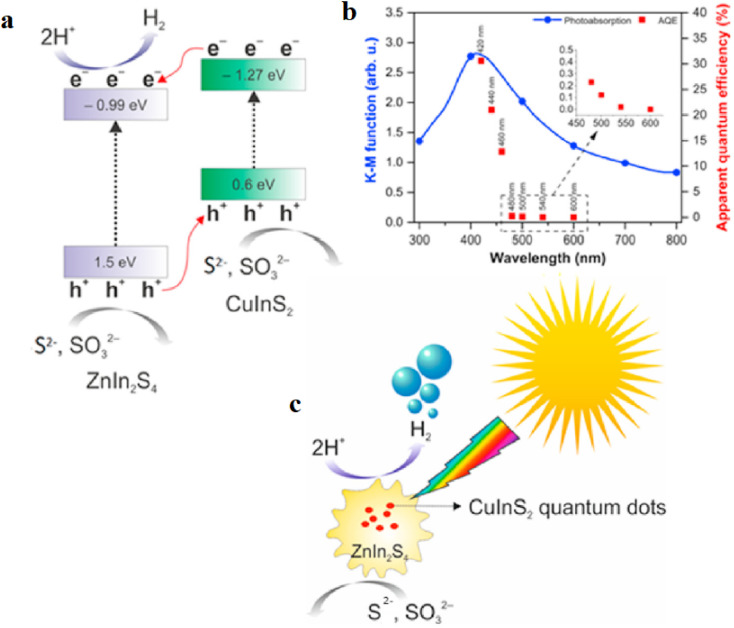
(a) Band alignment and interfacial charge-transfer pathways, (b) wavelength-dependent photocatalytic response and apparent quantum efficiency, and (c) schematic illustration of photocatalytic hydrogen evolution in the QD-based heterostructure. Reproduced with permission from ref. 66. © 2021 Elsevier B.V.

Interfacial charge-transfer processes play a decisive role in determining the photocatalytic efficiency of QD-based heterostructures. As shown in Fig. 4a, the band alignment between the constituent semiconductors establishes energetically favorable pathways for carrier migration, enabling photogenerated electrons to transfer toward the Pt cocatalyst while holes are extracted through the complementary semiconductor phase. This spatial separation suppresses electron–hole recombination and promotes efficient utilization of photoexcited carriers. The action spectrum presented in Fig. 4b further reveals a strong correlation between the wavelength-dependent photoresponse and hydrogen evolution activity, indicating that photocatalytic performance is governed not only by light absorption but also by the effectiveness of interfacial carrier transfer. Moreover, the mechanistic illustration in Fig. 4c summarizes the sequence of interfacial processes involved in photocatalytic hydrogen production, including charge generation, directional carrier transport, surface redox reactions, and hydrogen evolution at active catalytic sites. Collectively, these observations demonstrate that the interface functions as the central platform for regulating energy redistribution and charge-transfer pathways, thereby controlling the overall photocatalytic behavior of QD-based systems.

A notable trend is the growing appreciation of interface quality as a governing parameter. Photocatalytic performance is frequently limited not by intrinsic material properties but by poorly controlled interfacial environments that introduce energetic barriers, trap states, or unfavorable carrier pathways. This observation challenges the common strategy of focusing primarily on the optimization of individual material components while neglecting interface engineering. Nevertheless, the field continues to face substantial mechanistic ambiguity. Similar performance enhancements are often attributed to different transfer mechanisms, partly because direct experimental observation of interfacial processes remains difficult. The lack of universally accepted descriptors further complicates comparison among studies and hinders the development of predictive design frameworks. These considerations suggest that future advances will require a transition from material-centered design toward interface-centered design.^68–70^ Such a shift naturally raises a broader question: how can QDs are rationally integrated into increasingly complex photocatalytic architectures? Addressing this issue requires examining the functional roles that QDs assume within multi-component systems.

### 3.4. Functional integration of QDs in advanced photocatalytic architectures

The role of QDs in photocatalytic systems extends far beyond that of passive light absorbers. Increasingly, QDs are being incorporated as multifunctional components capable of simultaneously influencing light harvesting, charge management, interfacial energetics, and reaction environments. This evolution reflects a broader conceptual shift within photocatalysis, where performance is no longer viewed as the sum of individual material properties but as an emergent outcome of coordinated interactions among system components.

Current research trends reveal a progressive movement from single-component photocatalysts toward hierarchically organized architectures. Within these systems, QDs may serve as sensitizers, charge mediators, energy-transfer centers, electronic modulators, or structural regulators depending on the design strategy employed. Importantly, these roles are not mutually exclusive. A single QD population may participate in several functions simultaneously, creating synergistic effects that cannot be predicted from isolated material characteristics. However, increasing architectural complexity introduces new challenges.^71–73^ Enhanced performance is frequently reported without a clear understanding of which functional role is primarily responsible for the observed improvement. As a result, mechanistic interpretations often become speculative, limiting the transferability of design concepts across different photocatalytic platforms. This issue is particularly significant because many reported architectures are optimized empirically rather than developed through predictive principles.

**Fig. 5:**
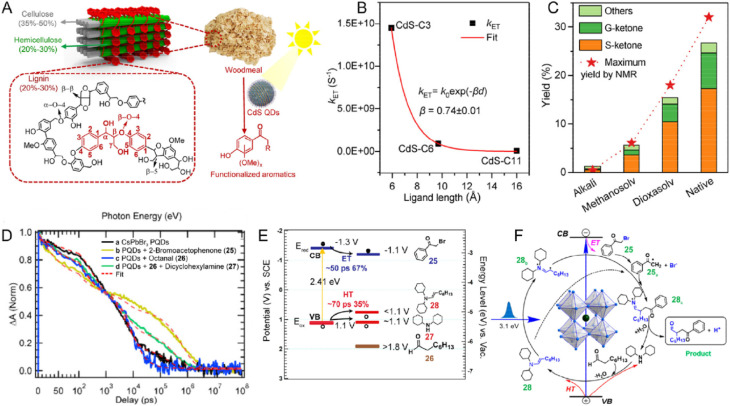
(A) Photocatalytic lignin valorization by CdS QDs, (B) ligand-length-dependent electron transfer, (C) catalytic performance for lignin conversion. Reproduced with permission from ref. 72. © 2019 American Chemical Society. (D) Transient absorption kinetics of perovskite QDs, (E) energy-level alignment, and (F) proposed photocatalytic reaction mechanism. Reproduced with permission from ref. 73. © 2020 American Chemical Society.

Fig. 5 highlights the multifunctional integration of QDs within advanced photocatalytic architectures, where their role extends beyond light absorption to include charge-transfer mediation, interfacial regulation, and catalytic activation. As shown in Fig. 5A, CdS QDs are incorporated into biomass valorization systems for the selective photocatalytic depolymerization of lignin, demonstrating their ability to drive complex chemical transformations in heterogeneous reaction environments. The relationship between electron-transfer rate and ligand length presented in Fig. 5B reveals the critical role of surface engineering in regulating interfacial charge transport, while the photocatalytic yields shown in Fig. 5C further illustrate how efficient carrier transfer can directly influence catalytic performance. Additional insight into the multifunctional behavior of QDs is provided by the perovskite QD system. The transient absorption kinetics in Fig. 5D demonstrate the formation of long-lived charge-separated states following photoexcitation, highlighting the ability of QDs to manage charge-carrier dynamics within integrated photocatalytic platforms. The energy-level alignment shown in Fig. 5E establishes favorable pathways for electron and hole transfer between QDs and reactants, whereas the mechanistic scheme in Fig. 5F illustrates how these photogenerated carriers participate in selective bond-forming reactions. Collectively, these results demonstrate that QDs can simultaneously function as light harvesters, charge mediators, interfacial regulators, and catalytic centers, emphasizing their central role in the design of increasingly sophisticated photocatalytic architectures.

Importantly, it is necessary to distinguish between quantum confinement-induced effects and general nanoscale phenomena in QD-based photocatalytic systems. While Section 3.1–3.3 establish that quantum confinement governs discrete electronic states and excited-state evolution, many performance enhancements reported in practical systems arise predominantly from interfacial charge separation, Schottky barrier formation, and plasmonic excitation. Therefore, in the following Section 4, each application case is critically evaluated to explicitly differentiate true quantum confinement contributions (*e.g.*, energy level discretization, tunable hot-electron distribution, and size-dependent electronic transitions) from conventional nanoscale effects that are not uniquely characteristic of quantum dots. This classification provides a consistent mechanistic framework for interpreting AgQD performance across different catalytic environments.

Table 2 summarizes the fundamental mechanisms governing quantum-dot-mediated photocatalysis and highlights the transition from photon absorption to catalytic functionality. The framework demonstrates that photocatalytic performance is controlled by a sequence of interconnected processes, including quantum-confined excitation, charge-carrier evolution, interfacial energy transfer, and functional integration within complex architectures. Rather than acting solely as light absorbers, QDs function as multifunctional modules that regulate energy flow, charge utilization, and catalytic activation. These insights provide a mechanistic foundation for the rational design of advanced photocatalytic systems.

**Table 2:** Unified mechanistic-to-design framework for QD photocatalysis

Mechanistic domain	Core phenomenon	Key descriptor	Role in photocatalysis	Main limitation	Design strategy
Quantum excitation	Discrete electronic transitions	Energy level quantization	Photon utilization efficiency	Incomplete excited-state description	Quantum-state engineering
Optical response	Competing light–matter processes	Electronic structure distribution	Selective absorption behavior	Mechanistic overlap (plasmon *vs.* quantum)	Electronic structure tuning
Carrier dynamics	Relaxation and redistribution	Density of states	Energy loss *vs.* utilization balance	Unclear relaxation pathways	Excited-state control
Charge generation	Photocarrier formation	Confinement strength	Driving force for reactions	Generation–utilization mismatch	Optimized size regime
Charge separation	Spatial carrier splitting	Interfacial coupling	Reduced recombination	Limited separation efficiency	Heterostructure design
Charge utilization	Surface reaction kinetics	Active surface sites	Catalytic conversion efficiency	Poor carrier extraction	Surface engineering
Recombination	Radiative/non-radiative decay	Defect density	Controls efficiency/selectivity	Oversimplified interpretation	Defect management
Interface transfer	Electron/hole migration	Energy alignment	Surface reaction activation	Transfer resistance	Interface engineering
Energy transfer	Excitonic + non-radiative processes	Coupling strength	Expanded reaction pathways	Mechanistic ambiguity	Interfacial coupling design
System integration	Multi-component architectures	System hierarchy	Synergistic enhancement	Functional role ambiguity	Modular design strategy
Architecture scaling	Hierarchical organization	System-level structure	Overall performance optimization	Lack of predictive descriptors	Rational system design

A critical knowledge gap therefore concerns the establishment of functional descriptors capable of quantitatively defining the role of QDs within integrated systems. Such descriptors would facilitate comparisons across studies and accelerate the transition from trial-and-error optimization toward rational engineering approaches.^74,75^ From this perspective, QDs should be viewed not merely as nanomaterials but as functional modules whose behavior depends strongly on system-level interactions. This framework provides the conceptual bridge to Section 4, where these mechanistic principles are examined in the context of specific photocatalytic reactions and practical application scenarios.

## 4. AgQD photocatalysts in practice: progress in hydrogen evolution, CO_2_ reduction, and related photocatalytic applications

### 4.1. AgQDs for photocatalytic hydrogen evolution: from plasmonic sensitization to charge management

Hydrogen evolution represents one of the most extensively explored application domains for AgQD-based photocatalysts. However, the available studies indicate that the contribution of AgQDs extends beyond the conventional role of plasmonic photosensitizers. Instead, AgQDs appear to function as multifunctional catalytic modules capable of simultaneously modulating light harvesting, charge separation, electron storage, and interfacial charge-transfer processes. This multifunctionality largely explains why performance improvements frequently exceed what would be expected from optical enhancement alone.^53,70^

The integration of AgQDs with hierarchical TiO_2_ nanotube architectures provides an illustrative example of this behavior. When AgQDs with tunable sizes ranging from 1.3 to 21.0 nm were deposited onto hierarchical TiO_2_ nanotube arrays, the resulting photoelectrodes exhibited markedly enhanced visible-light absorption, increased photocurrent density, and superior photoelectrocatalytic hydrogen evolution activity under visible-light irradiation.^76^ Importantly, the observed enhancement was attributed not only to SPR but also to the unique anti-shielding effect of ultrasmall AgQDs and the hierarchical architecture that facilitated carrier transport. This observation suggests that structural integration may be as important as plasmonic excitation itself. In this system, the enhanced performance is mainly governed by surface plasmon resonance and heterostructure-assisted charge transport within the TiO_2_ framework. The contribution of quantum confinement is indirect, primarily influencing the electronic properties of ultrasmall Ag domains, while the dominant mechanism is interfacial charge separation rather than discrete energy-level effects.

**Fig. 6:**
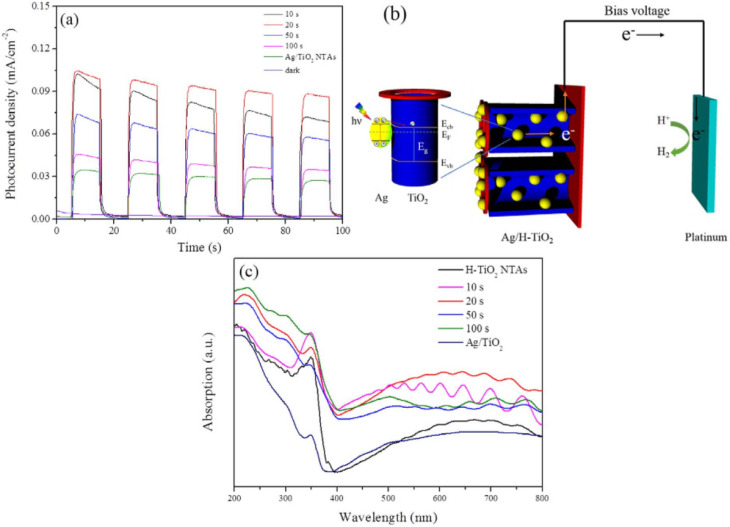
(a) Photocurrent responses of Ag QD-modified hierarchical TiO_2_ nanotube arrays under visible-light irradiation, (b) SPR-induced charge-transfer mechanism and photocatalytic hydrogen evolution pathway, and (c) diffuse reflectance spectra showing enhanced visible-light absorption after Ag QD incorporation. Reproduced from ref. 76 under the Creative Commons Attribution (CC BY) License. © Nature.

Fig. 6 demonstrates how Ag QDs function as multifunctional components that simultaneously enhance light harvesting, charge separation, and electron transport during photocatalytic hydrogen evolution. As shown in Fig. 6a, Ag QD-modified hierarchical TiO_2_ nanotube arrays exhibited substantially higher photocurrent densities under visible-light irradiation compared with bare TiO_2_ and conventional Ag/TiO_2_ systems, indicating more efficient generation and separation of photogenerated charge carriers. The optimum photocurrent response observed for the 20 s Ag/H–TiO_2_–NTAs sample suggests the existence of a balance between plasmonic enhancement and structural accessibility, whereas excessive Ag deposition leads to light-shielding effects and reduced photoactivity. The charge-transfer mechanism proposed in Fig. 6b further illustrates the multifunctional role of Ag QDs. Upon visible-light excitation, surface plasmon resonance generates energetic electrons that overcome the Ag/TiO_2_ Schottky barrier and are injected into the TiO_2_ conduction band, where the hierarchical nanotube architecture facilitates rapid electron transport toward hydrogen evolution sites. Simultaneously, the remaining positive charges on Ag QDs participate in oxidation reactions, thereby suppressing charge recombination and improving overall carrier utilization. Consistent with these observations, the diffuse reflectance spectra shown in Fig. 6c reveal a pronounced enhancement of visible-light absorption after Ag QD incorporation, with the strongest plasmonic response observed for the optimally loaded sample. Collectively, these results demonstrate that the superior hydrogen evolution performance originates not only from enhanced plasmonic light absorption but also from efficient charge management enabled by the synergistic integration of ultrasmall Ag QDs and hierarchical TiO_2_ architectures.

A similar conclusion emerges from AgQD/g-C_3_N_4_ systems. An optimum Ag loading of 0.02 mol generated a hydrogen evolution rate of 335.4 µmol g^−1^ h^−1^, substantially outperforming both pristine g-C_3_N_4_ and samples with higher Ag contents. The existence of a loading optimum highlights a recurring trend across AgQD photocatalysis: increasing Ag content does not necessarily improve performance.^77^ Excessive loading can introduce light-screening effects, agglomeration, or additional recombination pathways that offset plasmonic benefits.

Comparison of these studies reveals that efficient hydrogen production is governed by a balance between photon harvesting and charge management. While SPR-driven excitation enhances visible-light utilization, sustained catalytic activity depends on the ability of AgQDs to suppress electron–hole recombination and facilitate directional electron migration. Consequently, future design strategies should focus less on maximizing plasmonic intensity and more on engineering synergistic interfaces that simultaneously optimize optical and electronic functions. This shift from plasmon-centered design to charge-management-centered design appears to be a defining trend in the evolution of AgQD-based hydrogen evolution photocatalysts.

**Fig. 7:**
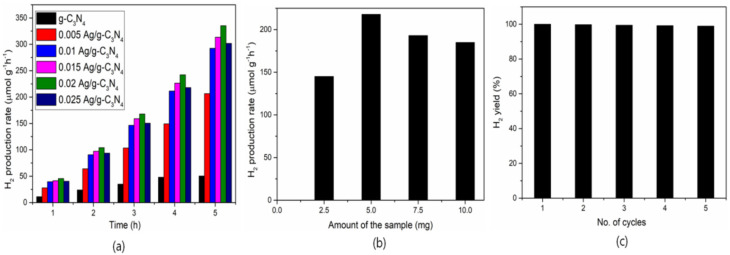
(a) Hydrogen evolution performance of Ag QD-modified g-C_3_N_4_ with different Ag loadings, (b) effect of catalyst dosage on hydrogen production, and (c) recyclability and stability of the optimized photocatalyst during repeated hydrogen evolution cycles. Reproduced from ref. 77 under the Creative Commons Attribution (CC BY) License. © 2021 MDPI.

Fig. 7 demonstrates the critical role of Ag QDs in regulating photocatalytic hydrogen evolution through both plasmonic enhancement and charge-management effects. As shown in Fig. 7a, the incorporation of Ag QDs into g-C_3_N_4_ substantially increased hydrogen production under visible-light irradiation compared with pristine g-C_3_N_4_, confirming the beneficial contribution of Ag-induced charge separation and improved utilization of photogenerated electrons. Notably, the hydrogen evolution activity exhibited a strong dependence on Ag loading, with the 0.02 Ag–g-C_3_N_4_ samples achieving the highest hydrogen production rate. This optimum behavior suggests that photocatalytic performance is governed by a balance between enhanced electron harvesting and adverse effects associated with excessive Ag content. At low loadings, Ag QDs promote efficient electron trapping and suppress electron–hole recombination, whereas excessive Ag deposition may introduce light-screening effects and block catalytically active sites, thereby reducing overall activity. The influence of catalyst dosage is further illustrated in Fig. 7b, where hydrogen generation varies with photocatalyst loading, emphasizing the importance of optimizing both material composition and reaction conditions. In addition, the recycling results presented in Fig. 7c reveal only a slight decline in hydrogen production after repeated reaction cycles, indicating good structural stability and sustained photocatalytic functionality. Collectively, these observations support the view that the superior performance of AgQD-modified g-C_3_N_4_ arises not simply from enhanced light absorption but from effective charge management, optimized interfacial electron transfer, and the establishment of an appropriate Ag loading window for sustained hydrogen evolution.

### 4.2. AgQDs for photocatalytic CO_2_ reduction: emerging evidence for direct plasmon-driven catalysis

Among the diverse photocatalytic applications of AgQDs, CO_2_ reduction occupies a particularly significant position because it directly tests the ability of photocatalysts to drive thermodynamically demanding multi-electron reactions. Interestingly, the available literature suggests that AgQDs may not simply function as auxiliary cocatalysts coupled to semiconductors, but can themselves become the primary photocatalytic reaction centers under solar irradiation. This proposition challenges one of the dominant assumptions in photocatalysis, namely that semiconductors necessarily constitute the active sites while noble metals serve only as electron sinks or plasmonic enhancers.^66,73^

A notable example is provided by AgQD-supported photocatalysts capable of utilizing the entire visible solar spectrum through plasmonic excitation. Mechanistic investigations demonstrated that AgQDs acted as the actual photocatalytic centers, while the direction of plasmon-driven charge transfer depended strongly on the nature of the support material.^78^ Remarkably, modifying the support altered whether AgQDs functioned predominantly as oxidation-active or reduction-active sites. This finding highlights an often-overlooked principle: the photocatalytic role of AgQDs is not intrinsic but is defined by interfacial electronic interactions.

Another important observation concerns the reaction pathway itself. Unlike many semiconductor systems that generate highly reactive radical intermediates, AgQDs were reported to promote electron tunneling interactions with adsorbed molecules, facilitating CO_2_ conversion through alternative charge-transfer routes. Such behavior was associated with efficiency improvements of several orders of magnitude compared with conventional photocatalytic processes. Although these results are highly promising, they also raise unresolved questions regarding the universality of the proposed mechanism and its dependence on catalyst architecture.^57,63^

From a broader perspective, current evidence indicates that AgQD-mediated CO_2_ reduction is evolving from a plasmon-enhanced semiconductor paradigm toward a direct plasmonic catalysis paradigm. Nevertheless, mechanistic validation remains limited, and quantitative comparisons among different AgQD systems are still scarce. Future progress therefore requires establishing clearer relationships between plasmon-induced charge transfer, surface reaction pathways, and product selectivity. Such understanding will be essential for translating laboratory-scale demonstrations into practical solar-fuel generation technologies.

**Fig. 8:**
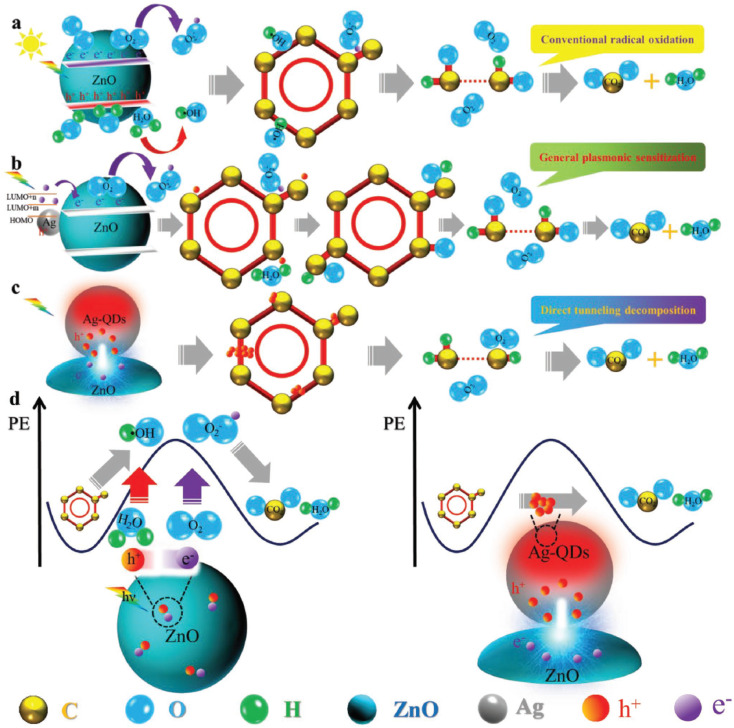
Comparison of photocatalytic mechanisms: (a) radical-mediated semiconductor photocatalysis, (b) plasmon-sensitized charge transfer in Ag/semiconductor systems, (c) direct plasmon-driven electron tunneling catalysis over AgQDs, and (d) energetic comparison between radical and tunneling reaction pathways. Reproduced from ref. 78 under the Creative Commons Attribution (CC BY) License. © 2023 Wiley-VCH GmbH. Frontiersin, MDPI, Nature.

Fig. 8 provides a mechanistic comparison between conventional semiconductor photocatalysis, plasmon-sensitized photocatalysis, and the emerging direct plasmon-driven catalytic behavior of AgQDs. In panel (a), the photocatalytic oxidation pathway in ZnO proceeds predominantly through the generation of reactive oxygen species (˙OH and ˙O_2_^−^), which subsequently oxidize organic molecules *via* multistep radical-mediated reactions. Panel (b) illustrates the plasmon-sensitization mechanism of Ag/ZnO, where plasmon-generated hot electrons are injected into the semiconductor, while the remaining holes exhibit limited oxidation capability, resulting in slower degradation kinetics and accumulation of intermediate species. In contrast, panel (c) proposes a fundamentally different mechanism for Ag-QDs/ZnO, where AgQDs act as the primary photoactive centers. Upon plasmon excitation, surface charges directly participate in coupled proton–electron transfer processes, enabling direct oxidation of adsorbed molecules without relying on free-radical intermediates. The energetic implications of these pathways are further highlighted in panel (d), where electron tunneling through AgQDs allows reactions to bypass the high-energy barriers associated with conventional radical routes. This mechanistic transition from plasmon-enhanced semiconductor photocatalysis toward direct plasmonic catalysis supports the emerging view that AgQDs can function not merely as electron mediators but as active photocatalytic centers capable of driving surface reactions through alternative charge-transfer pathways. Although plasmon-driven charge transfer and electron tunneling are key governing mechanisms in CO_2_ reduction, the specific contribution of quantum confinement is not independently isolated in most reported systems. The observed activity is therefore primarily attributed to plasmonic excitation and support-dependent interfacial electronic coupling rather than purely size-induced electronic discretization.

### 4.3. Environmental remediation through AgQD photocatalysts: water purification and pollutant degradation

Environmental remediation involving AgQD photocatalysts can be clearly divided into two mechanistically distinct pathways: (i) oxidative degradation of organic pollutants in aqueous media and (ii) photocatalytic disinfection of microbial contaminants. Although both processes are driven by photoinduced charge generation, the nature of reactive species and reaction channels differs fundamentally.

In the case of organic pollutant degradation, the dominant mechanism is the generation of reactive oxygen species (ROS), particularly ˙OH and ˙O_2_^−^ radicals, which oxidatively decompose dye molecules and pharmaceutical residues. For instance, AgQD-decorated SnO_2_ nanoflakes significantly enhance RhB degradation, achieving approximately twice the efficiency of pristine SnO_2_ due to improved interfacial electron transfer and Schottky junction formation that promotes ROS generation.^79^ Similarly, AgQDs embedded in CTS–PEO polymer matrices achieve 91.1% degradation of *p*-nitrophenol within 3 h, where polymer confinement improves surface accessibility and stabilizes interfacial charge separation pathways.^80^ In these systems, AgQDs primarily act as electron mediators that enhance ROS production rather than directly participating in molecular bond cleavage.

In contrast, photocatalytic disinfection systems involve biological interaction channels dominated by membrane disruption and intracellular damage. In AgQD/Bi_4_O_5_Br_2_ composites, complete inactivation of *E. coli* (≈1 × 10^7^ cfu mL^−1^) is achieved under visible light irradiation.^81^ Here, ROS species together with photogenerated holes induce oxidative stress on bacterial cell walls, leading to leakage of intracellular components and irreversible membrane damage. Unlike organic oxidation, this pathway involves complex bio–interface interactions, where electron tunneling and surface redox processes contribute to cellular destruction. Overall, AgQDs play a dual but mechanistically differentiated role: ROS-driven chemical oxidation in pollutant degradation *versus* ROS-assisted bioelectrical disruption in antimicrobial processes. This distinction is critical for accurate mechanistic interpretation.

**Fig. 9:**
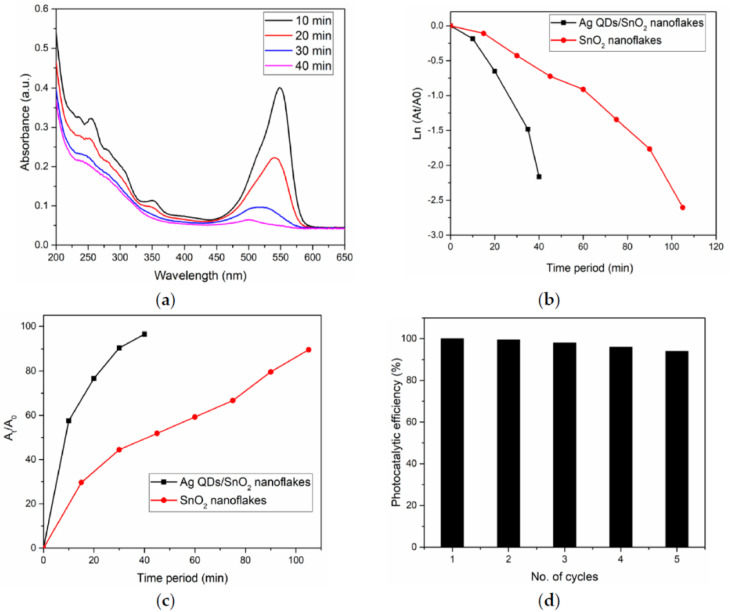
Photocatalytic RhB degradation over Ag QDs/SnO_2_ nanoflakes: (a) degradation efficiency, (b) pseudo-first-order kinetics, (c) time-dependent concentration profile, and (d) catalyst recyclability. Reproduced from ref. 79 under the Creative Commons Attribution (CC BY) License. © 2019 MDPI.

As shown in Fig. 9a, Ag QDs/SnO_2_ exhibits significantly enhanced RhB degradation under light irradiation compared with bare SnO_2_, confirming the beneficial role of Ag QDs in improving photocatalytic efficiency. The kinetic behavior is further analyzed in Fig. 9b, where a linear relationship between ln(At/A0) and irradiation time confirms that the degradation follows a pseudo-first-order Langmuir–Hinshelwood model. The higher apparent rate constant (*k*) observed for Ag QDs/SnO_2_ compared to SnO_2_ indicates accelerated reaction kinetics induced by improved charge separation and interfacial electron transfer. The temporal evolution of RhB concentration during photocatalysis is presented in Fig. 9c, demonstrating a steady decrease in pollutant concentration with irradiation time. Finally, Fig. 9d illustrates the recyclability of the Ag QDs/SnO_2_ photocatalyst over five consecutive cycles, where only a slight decrease in degradation efficiency (≈94% in the fifth cycle) is observed, attributed to reduced surface interaction between reaction intermediates and the catalyst surface during repeated use. Overall, these results confirm that Ag QDs not only enhance photocatalytic activity but also contribute to maintaining reasonable structural stability and operational durability.

### 4.4. Expanding application frontiers: photoelectrochemical systems, air purification, and multifunctional photocatalysis

Recent advances demonstrate that AgQD photocatalysts operate across multiple application domains; however, each domain is governed by distinct charge-transfer and reaction channels. These include (i) photoelectrochemical charge extraction, (ii) gas-phase oxidative degradation, and (iii) multifunctional catalytic–antimicrobial processes.

In photoelectrochemical (PEC) systems, AgQDs primarily function as plasmonic sensitizers and charge extraction facilitators. For example, in hierarchical TiO_2_ nanotree arrays, AgQD incorporation extends light absorption into the visible range and enhances interfacial charge separation, resulting in a photocurrent density of approximately 0.52 mA cm^−2^, nearly 140 times higher than pristine TiO_2_.^82^ The governing channel in this system is directed electron transport through plasmon-assisted excitation and heterointerface-mediated charge migration.

In gas-phase photocatalysis, AgQDs embedded in Y-MOF structures act as electron reservoirs that regulate redox pathways for volatile organic compound (VOC) degradation. The system achieves an apparent quantum efficiency of 10.5% for acetaldehyde oxidation at 420 nm, corresponding to significant performance enhancement over pristine frameworks.^83^ The dominant channel here is ROS-mediated oxidation facilitated by charge accumulation and transfer at the AgQD–MOF interface.

In multifunctional systems, such as AgQD/g-C_3_N_4_ composites, multiple reaction channels coexist, including hydrogen evolution, organic dye degradation, and antimicrobial activity.^77^ However, these processes are not governed by a single unified mechanism. Instead, they arise from system-dependent channel selection, where photocatalytic oxidation (ROS pathways), reduction reactions (electron transfer), and biological interactions (membrane disruption) operate independently but are simultaneously enabled by AgQD-mediated charge modulation. Importantly, across all systems, AgQDs primarily act as interfacial charge mediators and plasmonic enhancers rather than universal active centers. The specific reaction outcome is determined by the dominant channel: oxidative chemistry in pollutants, bioelectrical disruption in disinfection, and directed electron flow in PEC systems.

The comparative analysis of AgQD-based photocatalytic systems (Table 3) clearly demonstrates that performance enhancement arises from multiple interrelated mechanisms rather than a single size-dependent effect. Across diverse platforms, including TiO_2_, g-C_3_N_4_, SnO_2_, MOFs, and polymeric matrices, AgQDs consistently contribute to improved light harvesting, charge separation, and interfacial electron transfer. Importantly, recent evidence indicates a functional transition from classical SPR sensitization toward direct plasmon-driven catalysis and electron reservoir behavior. These findings collectively suggest that AgQDs operate as multifunctional catalytic modules whose activity is governed by system architecture, interfacial coupling, and electronic structure rather than surface area alone or simple quantum confinement effects.

**Table 3:** Comparative performance and mechanistic roles of AgQDs in photocatalytic systems

Photocatalytic system	Role of AgQDs	Reaction	Key performance outcome	Dominant mechanism	Ref.
AgQDs/hierarchical TiO_2_ nanotubes	SPR sensitizer + electron injector	H_2_ evolution (PEC)	Strong visible absorption, up to ∼140× photocurrent increase	SPR + anti-shielding + charge separation	76
AgQDs/g-C_3_N_4_	Cocatalyst + charge mediator	H_2_ evolution + dye degradation	335.4 µmol g^−1^ h^−1^ H_2_ production	Charge separation + recombination suppression	77
AgQDs/supported systems	Primary catalytic center	CO_2_ reduction/oxidation	Orders-of-magnitude efficiency enhancement	Direct plasmon-driven electron tunneling	78
AgQDs/SnO_2_ nanoflakes	Electron acceptor + SPR enhancer	Rhodamine B degradation	∼2× higher degradation *vs.* SnO_2_	Schottky barrier + electron trapping	79
AgQDs/CTS–PEO polymer matrix	Stabilized photocatalytic centers	*p*-nitrophenol degradation	91.1% degradation in 3 h	SPR + band gap reduction + synergistic effects	80
AgQDs/Bi_4_O_5_Br_2_	Charge separation enhancer	Bacterial disinfection	100% *E. coli* elimination	ROS generation + SPR + Schottky barrier	81
AgQDs/TiO_2_ nanotree arrays	PEC sensitizer	Photoelectrochemical H_2_ evolution	0.52 mA cm^−2^, ∼140× enhancement	SPR + hierarchical charge transport	82
AgQDs/Y-MOF	Electron reservoir	VOC (acetaldehyde) degradation	AQE 10.5%, 20× improvement	Charge separation + ROS generation	83

### 4.5. From plasmonic additives to functional photocatalytic platforms: an emerging paradigm for AgQDs

A careful examination of the current literature reveals a significant conceptual shift in the role of AgQDs within photocatalytic systems. Early studies largely interpreted AgQDs as plasmonic sensitizers whose primary function was to extend light absorption into the visible region. However, the collective evidence from hydrogen production, CO_2_ reduction, pollutant degradation, disinfection, photoelectrochemical conversion, and VOC purification increasingly suggests that AgQDs should be viewed as multifunctional photocatalytic platforms rather than simple plasmonic additives. This emerging perspective may represent one of the most important developments in the AgQD photocatalysis field.

The most compelling evidence arises from the diversity of reaction environments in which AgQDs remain effective. In hydrogen evolution systems, AgQDs improve carrier separation and facilitate electron utilization, yielding hydrogen production rates as high as 335.4 µmol g^−1^ h^−1^ under visible light.^77^ In photoelectrochemical architectures, incorporation of AgQDs increases photocurrent generation by up to 140-fold relative to pristine TiO_2_ structures.^82^ Meanwhile, in CO_2_ photoreduction, AgQDs have been proposed to function as the actual photocatalytic active centers, enabling reaction efficiencies several orders of magnitude higher than conventional photocatalytic routes through plasmon-driven charge-transfer processes.^78^ Such observations challenge the traditional semiconductor-centered view of photocatalysis and suggest that AgQDs can directly participate in catalytic transformations.

An equally important trend is the remarkable adaptability of AgQDs across different material platforms. Enhanced activity has been demonstrated in metal oxides (TiO_2_ and SnO_2_),^76,82^ polymeric matrices,^80^ carbon nitride frameworks,^77^ bismuth oxyhalides,^81^ and metal–organic frameworks.^46^ Despite the structural diversity of these hosts, performance improvements are consistently associated with three recurring functions of AgQDs: promotion of visible-light harvesting, suppression of charge recombination, and modulation of interfacial electron transfer. The persistence of these functions across fundamentally different architectures suggests that they may represent universal design principles rather than system-specific phenomena.

Nevertheless, the field faces a critical mechanistic challenge. Similar performance enhancements are frequently attributed to SPR effects, Schottky barrier formation, electron-reservoir behavior, or direct catalytic activity. In many cases, these mechanisms are invoked simultaneously without clear experimental discrimination. The situation becomes even more complex when considering reports that AgQDs may mediate electron tunneling processes that bypass conventional free-radical pathways during CO_2_ conversion.^78^ Consequently, the literature currently contains a growing gap between observed performance gains and mechanistic certainty.^54,73^

From a broader perspective, the next frontier in AgQD photocatalysis is unlikely to be the discovery of new reactions. Instead, future progress will depend on establishing a unified framework capable of explaining why AgQDs exhibit such versatility across energy conversion, environmental remediation, and photoelectrochemical applications. The transition from reaction-specific optimization toward function-oriented design represents a highly visible research trend and may ultimately define the next generation of AgQD-enabled photocatalytic technologies.^80^

## 5. Toward rationally engineered AgQD photocatalysts: unresolved challenges, emerging design rules, and future research frontiers

### 5.1. Moving beyond performance metrics: the need for mechanistic certainty

Despite the rapid expansion of AgQD-based photocatalysis, mechanistic understanding has not progressed at the same pace as performance improvement. A substantial portion of the literature remains dominated by activity-oriented reporting, where enhanced hydrogen production, pollutant degradation, or CO_2_ conversion efficiencies are presented as indicators of success without establishing definitive causal relationships between structure and function. Consequently, the field faces a growing imbalance between performance claims and mechanistic certainty.

One of the most persistent challenges concerns the interpretation of plasmonic effects. Enhanced photocatalytic activity is frequently attributed to localized surface plasmon resonance, yet direct evidence distinguishing plasmon-induced charge transfer from alternative processes such as defect-mediated transport, Schottky barrier formation, or local electric-field enhancement is often lacking. As AgQDs approach the quantum-confined regime, this ambiguity becomes even more significant because classical plasmonic descriptions become increasingly inadequate.^76,83^

Another challenge arises from the widespread reliance on *ex situ* characterization techniques. Structural and electronic properties are typically evaluated before or after photocatalytic reactions, whereas the active catalyst frequently undergoes dynamic restructuring under illumination. Consequently, the true catalytic state may differ substantially from the state being characterized. This discrepancy limits the reliability of many proposed mechanisms.

Future research must therefore prioritize *in situ* real-time conditions (*operando*) spectroscopy, time-resolved measurements, and multiscale computational modeling capable of tracking catalyst evolution under realistic conditions. More importantly, the field requires a transition from correlation-based interpretations toward causality-driven investigations. Understanding why AgQDs function as they do will ultimately be more valuable than continuously reporting incremental improvements in activity. Such mechanistic clarity represents the essential foundation upon which rational catalyst design can be established.

### 5.2. Atomic precision as the next design frontier

The majority of current AgQD photocatalysts are still described using ensemble-averaged parameters such as particle size, loading amount, or optical absorption characteristics. While these descriptors provide useful information, they fail to capture the atomic-level complexity that increasingly appears to govern photocatalytic functionality. As a result, the next stage of AgQD development is likely to be driven by atomic precision engineering rather than conventional nanostructure optimization. Recent advances in nanochemistry suggest that changing only a few surface atoms may significantly alter electronic distributions, charge-transfer pathways, and optical responses.^77,82^ This sensitivity reflects the fact that AgQDs occupy a regime where atomic arrangement becomes as important as overall composition. Consequently, catalysts possessing identical average dimensions may exhibit fundamentally different photocatalytic behavior due to differences in atomic ordering or surface coordination environments.

A critical limitation of current research is that most synthesis methods generate structurally heterogeneous AgQD populations. Such heterogeneity complicates mechanistic interpretation because observed properties represent averaged responses from multiple structural species. This issue partly explains why contradictory conclusions frequently emerge across studies. Without atomic-level control, extracting universal design principles remains difficult.

Future strategies should therefore focus on atomically defined AgQD systems with precisely controlled size, composition, and surface structures. Integration of advanced electron microscopy, synchrotron-based characterization, and first-principles simulations will be essential for establishing direct structure–property relationships. Furthermore, machine-learning-assisted materials discovery may accelerate identification of optimal atomic configurations that are difficult to predict experimentally. The transition from nanoscale engineering to atomic-scale engineering represents more than a methodological improvement; it marks a conceptual shift in how AgQD photocatalysts are designed. Such precision is likely to become a defining characteristic of next-generation photocatalytic materials.

### 5.3. Interface-centered design: reconsidering the active site concept

Traditional photocatalysis often treats active sites as intrinsic properties of individual materials. However, emerging evidence increasingly indicates that the functional behavior of AgQDs is determined less by isolated particles and more by the interfaces they form with surrounding environments. This observation suggests that future progress may depend on redefining the active site concept itself. In many AgQD-based systems, photocatalytic performance originates from interactions occurring at heterointerfaces rather than within the AgQDs alone. Charge separation, carrier migration, energy transfer, and adsorption processes are all strongly influenced by local interfacial energetics. Consequently, identical AgQDs may exhibit entirely different catalytic functions when integrated into different host materials. Such behavior highlights the limitations of material-centric design approaches.^78,81^

Current catalyst development often focuses on maximizing interfacial contact area. While beneficial in some cases, this strategy does not necessarily guarantee efficient charge transfer. The quality of the interface may be more important than its quantity. Structural disorder, interfacial defects, and unfavorable energy alignments can introduce recombination pathways that offset potential benefits associated with increased contact. A major knowledge gap concerns the lack of quantitative descriptors capable of predicting interfacial functionality. Existing parameters such as band alignment or work-function differences provide only partial insights into complex interfacial phenomena. More comprehensive descriptors incorporating electronic coupling, carrier dynamics, and local structural effects are needed.^77^ Future photocatalytic architectures will likely evolve toward deliberately engineered interfaces where electronic communication between AgQDs and supporting materials is controlled with atomic precision. Such an interface-centered perspective may enable the design of catalysts whose properties emerge from cooperative interactions rather than from individual components. This approach offers a promising pathway toward overcoming many of the performance limitations currently encountered in AgQD photocatalysis.

### 5.4. From laboratory demonstrations to technological deployment

Although AgQD photocatalysts have demonstrated impressive performance in laboratory-scale studies, their transition toward practical implementation remains uncertain. Most reported systems are evaluated under highly controlled conditions that differ substantially from real operating environments. Consequently, performance metrics obtained in academic studies cannot be directly translated into technological viability. One major challenge involves long-term stability. Under continuous illumination, AgQDs may undergo oxidation, aggregation, dissolution, or surface reconstruction, leading to progressive performance degradation. Yet durability assessments remain considerably less common than activity measurements. This imbalance creates uncertainty regarding the practical lifetime of many reported photocatalysts.

Scalability represents another critical obstacle. Synthesis methods that provide excellent structural control at laboratory scales may become economically impractical when translated to industrial production. Furthermore, many studies prioritize catalyst efficiency without considering manufacturing complexity, material utilization efficiency, or lifecycle sustainability.

Standardization also remains insufficient.^80,82^ Differences in light sources, reactor configurations, quantum-efficiency calculations, and performance reporting protocols frequently hinder meaningful comparisons among studies. As a result, identifying genuinely superior photocatalytic systems remains challenging.

Future research should therefore incorporate techno-economic analysis, lifecycle assessment, and standardized benchmarking alongside traditional performance evaluation. Hybrid experimental–computational approaches may further accelerate the identification of scalable catalyst designs. Ultimately, the successes of AgQD photocatalysis will not be determined solely by achieving record efficiencies but by developing systems that simultaneously satisfy performance, durability, sustainability, and economic requirements. The next decade is likely to witness a transition from proof-of-concept demonstrations toward deployment-oriented research, where practical feasibility becomes as important as scientific novelty.

### 5.5. Computational and AI-driven design of AgQD photocatalysts: from first-principles modeling to data-driven discovery

Recent advances in computational chemistry and artificial intelligence are redefining the design paradigm of AgQD-based photocatalysts. In particular, first-principles methods such as density functional theory (DFT) and time-dependent DFT (TDDFT) provide a powerful framework for resolving size-dependent electronic structures, optical transitions, and excited-state dynamics in quantum-confined silver systems. These approaches enable direct quantification of how particle size, surface coordination, and ligand environment influence energy-level discretization, plasmonic damping, and hot-electron generation. Unlike conventional band-structure approximations, TDDFT-based simulations capture the coexistence of localized electronic states and collective plasmon-like excitations, which is essential for accurately describing ultrasmall AgQDs in the transitional regime between metallic and molecular behavior.

Beyond atomistic modeling, machine learning (ML) approaches are increasingly being integrated into nanomaterials research to accelerate structure–property discovery. By training models on computational and experimental datasets, ML algorithms can identify hidden correlations between structural descriptors (*e.g.*, size, defect density, ligand type) and functional outputs such as photocatalytic rate constants or charge-transfer efficiency.^78–81^ This enables rapid screening of large AgQD compositional spaces that would be computationally prohibitive using conventional simulations alone. Importantly, feature-importance analysis in ML models can also reveal dominant physical descriptors governing performance, thereby providing interpretability alongside prediction.

In the near future, the convergence of DFT/TDDFT and ML is expected to enable closed-loop materials discovery platforms, where candidate AgQD structures are computationally generated, screened for optimal optical–electronic properties, and experimentally validated in an iterative workflow. Additional milestones include the development of operando-validated quantum models, ligand-resolved electronic structure databases, and transferable ML potentials for plasmonic nanostructures. Collectively, these advances will shift AgQD photocatalyst design from empirical optimization toward fully predictive, data-driven engineering guided by quantum-level understanding.

### 5.6. Frontier research directions and integrated design roadmap for AgQD photocatalysis

The next stage of AgQD photocatalysis research will require a transition from performance-driven studies toward predictive, mechanism-guided, and system-level design frameworks. Current evidence indicates that catalytic performance is governed by a coupled interplay between quantum confinement, interfacial charge transfer, defect chemistry, and plasmon-mediated excitation processes. However, these effects are still not fully decoupled experimentally, highlighting the need for more structured research directions.

In basic mechanistic understanding, the key challenge is to resolve the coexistence of plasmonic and quantum-confined excitations under realistic reaction conditions. Emerging systems such as Zn QD-modified ZnWO_4_ with oxygen vacancies demonstrate that defect-assisted charge trapping and quantum nanodomains can synergistically enhance redox activity.^84^ Similarly, Ag-based heterostructures highlight the importance of distinguishing between plasmon-driven excitation and interface-mediated electron migration, which remain experimentally intertwined in most systems.^85^

From a synthesis and preparation perspective, achieving atomically controlled AgQDs with narrow size distribution and engineered surface chemistry remains a central target. Recent heterostructure strategies such as Ag/AgCl and g-C_3_N_4_-based composites demonstrate that precise control over nanoparticle dispersion and interfacial contact directly influences charge-transfer efficiency and photostability.^86^

In the domain of interface engineering, rational design of multi-component architectures (*e.g.*, ZnWO_4_/AgCl systems) reveals that synergistic coupling between plasmonic Ag species and semiconductor supports enables multifunctional performance including photocatalysis, antibacterial activity, and antifouling behavior.^87^ These findings emphasize that interface quality, rather than simple contact area, is the dominant factor controlling catalytic efficiency.

Finally, for device-level translation, future research should prioritize scalable architectures, *operando* characterization, and machine-learning-assisted catalyst screening. Integration of *in situ* spectroscopy with data-driven modeling is expected to enable predictive optimization of AgQD systems within the next 3–5 years. Overall, these directions collectively define a unified roadmap toward rational, scalable, and application-ready AgQD photocatalysts.

## 6. Conclusion

Silver quantum dots (AgQDs) represent a transformative class of nanoscale photocatalysts in which classical plasmonic behavior gradually evolves into quantum-confined electronic regimes. This duality endows AgQDs with properties that extend beyond conventional noble-metal nanoparticles, enabling tunable electronic structure, interface-sensitive reactivity, and non-classical light–matter interactions. However, the most important insight emerging from this review is that the photocatalytic performance of AgQDs is not governed by a single dominant mechanism, but rather by a dynamic balance between quantum confinement effects, plasmonic excitation behavior, and interfacial charge-transfer processes. A critical analysis of recent literature reveals that the enhancement mechanisms often attributed to AgQDs are frequently interdependent and context-specific. In particular, the same material may exhibit plasmon-driven, interface-controlled, or quantum-modulated behavior depending on particle size, surface chemistry, and coupling environment. This underscores the need to move beyond descriptive interpretations toward unified mechanistic frameworks capable of distinguishing and quantifying these contributions.

Despite significant progress, the field remains constrained by the lack of atomic-level structural control, limited *operando* mechanistic understanding, and insufficient predictive modeling of structure–function relationships. These limitations currently prevent the establishment of universal design principles for AgQD photocatalysis. Future progress will depend on integrating atomic precision synthesis, *operando* spectroscopic validation, and data-driven computational approaches to enable predictive catalyst engineering. In particular, interface-first design strategies and multiscale modeling frameworks are expected to play a central role in translating AgQD properties into controllable catalytic functions. Ultimately, advancing this field requires shifting from performance-centric reporting toward mechanism-driven and design-oriented photocatalytic science.

## Conflicts of interest

The authors declare that they have no known competing financial interests or personal relationships that could have appeared to influence the work reported in this paper.

## Data Availability

No primary research results, software or code have been included and no new data were generated or analysed as part of this review.
